# Cross-trait analyses with migraine reveal widespread pleiotropy and suggest a vascular component to migraine headache

**DOI:** 10.1093/ije/dyaa050

**Published:** 2020-04-19

**Authors:** Katherine M Siewert, Derek Klarin, Scott M Damrauer, Kyong-Mi Chang, Philip S Tsao, Themistocles L Assimes, George Davey Smith, Benjamin F Voight, The International Headache Genetics Consortium, Padhraig Gormley, Verneri Anttila, Bendik S Winsvold, Priit Palta, Tonu Esko, Tune H. Pers, Kai-How Farh, Ester Cuenca-Leon, Mikko Muona, Nicholas A Furlotte, Tobias Kurth, Andres Ingason, George McMahon, Lannie Ligthart, Gisela M Terwindt, Mikko Kallela, Tobias M Freilinger, Caroline Ran, Scott G Gordon, Anine H Stam, Stacy Steinberg, Guntram Borck, Markku Koiranen, Lydia Quaye, Hieab HH Adams, Terho Lehtimäki, Antti-Pekka Sarin, Juho Wedenoja, David A Hinds, Julie E Buring, Markus Schürks, Paul M Ridker, Maria Gudlaug Hrafnsdottir, Hreinn Stefansson, Susan M Ring, Jouke-Jan Hottenga, Brenda WJH Penninx, Markus Färkkilä, Ville Artto, Mari Kaunisto, Salli Vepsäläinen, Rainer Malik, Andrew C Heath, Pamela A F Madden, Nicholas G Martin, Grant W Montgomery, Mitja Kurki, Mart Kals, Reedik Mägi, Kalle Pärn, Eija Hämäläinen, Hailiang Huang, Andrea E Byrnes, Lude Franke, Jie Huang, Evie Stergiakouli, Phil H Lee, Cynthia Sandor, Caleb Webber, Zameel Cader, Bertram Muller-Myhsok, Stefan Schreiber, Thomas Meitinger, Johan G Eriksson, Veikko Salomaa, Kauko Heikkilä, Elizabeth Loehrer, Andre G Uitterlinden, Albert Hofman, Cornelia M van Duijn, Lynn Cherkas, Linda M. Pedersen, Audun Stubhaug, Christopher S Nielsen, Minna Männikkö, Evelin Mihailov, Lili Milani, Hartmut Göbel, Ann-Louise Esserlind, Anne Francke Christensen, Thomas Folkmann Hansen, Thomas Werge, Sigrid Børte, Bru Cormand, Else Eising, Lyn Griffiths, Eija Hamalainen, Marjo Hiekkala, Risto Kajanne, Lenore Launer, Terho Lehtimaki, Davor Lessel, Alfons Macaya, Massimo Mangino, Nancy Pedersen, Danielle Posthuma, Patricia Pozo-Rosich, Alice Pressman, Celia Sintas, Marta Vila-Pueyo, Huiying Zhao, Jaakko Kaprio, Arpo J Aromaa, Olli Raitakari, M Arfan Ikram, Tim Spector, Marjo-Riitta Järvelin, Andres Metspalu, Christian Kubisch, David P Strachan, Michel D Ferrari, Andrea C Belin, Martin Dichgans, Maija Wessman, Arn MJM van den Maagdenberg, John-Anker Zwart, Dorret I Boomsma, George Davey Smith, Kari Stefansson, Nicholas Eriksson, Mark J Daly, Benjamin M Neale, Jes Olesen, Daniel I Chasman, Dale R Nyholt, Aarno Palotie

**Affiliations:** 111 Genomics and Computational Biology Graduate Group, Perelman School of Medicine, University of Pennsylvania, Philadelphia, PA, USA; 112 Center for Genomic Medicine, Massachusetts General Hospital, Harvard Medical School, Boston, MA, USA; 113 Program in Medical and Population Genetics, Broad Institute of MIT and Harvard, Cambridge, MA, USA; 114 Boston VA Healthcare System, Boston, MA, USA; 115 Corporal Michael Crescenz VA Medical Center, Philadelphia, PA, USA; 116 Department of Surgery, Perelman School of Medicine, University of Pennsylvania, Philadelphia, PA, USA; 117 Department of Medicine, Perelman School of Medicine, University of Pennsylvania, Philadelphia, PA, USA; 118 Department of Medicine, Stanford University School of Medicine, Stanford, CA, USA; 119 VA Palo Alto Health Care System, Palo Alto, CA, USA; 1110 Medical Research Council (MRC) Integrative Epidemiology Unit, University of Bristol, Bristol, UK; 1111 Bristol Medical School, Population Health Sciences, University of Bristol, Bristol, UK; 1112 Department of Systems Pharmacology and Translational Therapeutics, Perelman School of Medicine, University of Pennsylvania, Philadelphia, PA, USA; 1113 Department of Genetics, Perelman School of Medicine, University of Pennsylvania, Philadelphia, PA, USA; 1114 Institute for Translational Medicine and Therapeutics, Perelman School of Medicine, University of Pennsylvania, Philadelphia, PA, USA; 1 Psychiatric and Neurodevelopmental Genetics Unit, Massachusetts General Hospital and Harvard Medical School, Boston, USA; 2 Medical and Population Genetics Program, Broad Institute of MIT and Harvard, Cambridge, USA; 3 Stanley Center for Psychiatric Research, Broad Institute of MIT and Harvard, Cambridge, USA; 4 Wellcome Trust Sanger Institute, Wellcome Trust Genome Campus, Hinxton, UK; 5 Analytic and Translational Genetics Unit, Massachusetts General Hospital and Harvard Medical School, Boston, USA; 6 FORMI, Oslo University Hospital, P.O. 4956 Nydalen, 0424 Oslo, Norway; 7 Department of Neurology, Oslo University Hospital, P.O. 4956 Nydalen, 0424 Oslo, Norway; 8 Institute of Clinical Medicine, University of Oslo, P.O. 1171 Blindern, 0318 Oslo, Norway; 9 Institute for Molecular Medicine Finland (FIMM), University of Helsinki, Helsinki, Finland; 10 Estonian Genome Center, University of Tartu, Tartu, Estonia; 11 Division of Endocrinology, Boston Children's Hospital, Boston, USA; 12 Statens Serum Institut, Dept of Epidemiology Research, Copenhagen, Denmark; 13 Novo Nordisk Foundation Center for Basic Metabolic Research, University of Copenhagen, Copenhagen, Denmark; 14 Illumina, 5200 Illumina Way, San Diego, USA; 15 Vall d'Hebron Research Institute, Pediatric Neurology, Barcelona, Spain; 16 Folkhälsan Institute of Genetics, Helsinki, Finland, FI-00290; 17 Neuroscience Center, University of Helsinki, Helsinki, Finland, FI-00014; 18 Research Programs Unit, Molecular Neurology, University of Helsinki, Helsinki, Finland, FI-00014; 19 23andMe, Inc., 899 W. Evelyn Avenue, Mountain View, CA, USA; 20 Inserm Research Center for Epidemiology and Biostatistics (U897), University of Bordeaux, 33076 Bordeaux, France.; 21 Division of Preventive Medicine, Brigham and Women's Hospital, Boston MA 02215; 22 deCODE Genetics, 101 Reykjavik, Iceland.; 23 Medical Research Council (MRC) Integrative Epidemiology Unit, University of Bristol, Bristol, UK; 24 VU University Amsterdam, Department of Biological Psychology, Amsterdam, the Netherlands, 1081 BT.; 25 Leiden University Medical Centre, Department of Neurology, Leiden, The Netherlands, PO Box 9600, 2300 RC.; 26 Department of Neurology, Helsinki University Central Hospital, Haartmaninkatu 4, 00290 Helsinki, Finland; 27 Institute for Stroke and Dementia Research, Klinikum der Universtität München, Ludwig-Maximilians-Universität München, Feodor-Lynen-Str. 17, 81377 Munich Germany.; 28 Department of Neurology and Epileptology, Hertie Institute for Clincal Brain Research, University of Tuebingen.; 29 Karolinska Institutet, Department of Neuroscience, 171 77 Stockholm, Sweden; 30 Department of Genetics and Computational Biology, QIMR Berghofer Medical Research Institute, 300 Herston Road, Brisbane, QLD 4006, Australia; 31 Ulm University, Institute of Human Genetics, 89081 Ulm, Germany; 32 University of Oulu, Center for Life Course Epidemiology and Systems Medicine, Oulu, Finland, Box 5000, Fin-90014 University of Oulu.; 33 Department of Twin Research and Genetic Epidemiology, King's College London, London, UK; 34 Dept of Epidemiology, Erasmus University Medical Center, Rotterdam, the Netherlands, 3015 CN.; 35 Dept of Radiology, Erasmus University Medical Center, Rotterdam, the Netherlands, 3015 CN.; 36 Department of Clinical Chemistry, Fimlab Laboratories, and School of Medicine, University of Tampere, Tampere, Finland, 33520; 37 Department of Public Health, University of Helsinki, Helsinki, Finland; 38 Harvard Medical School, Boston MA 02115; 39 University Duisburg Essen, Essen, Germany; 40 Landspitali University Hospital, 101 Reykjavik, Iceland.; 41 VU University Medical Centre, Department of Psychiatry, Amsterdam, the Netherlands, 1081 HL.; 42 Department of Psychiatry, Washington University School of Medicine, 660 South Euclid, CB 8134,St. Louis, MO 63110, USA; 43 University Medical Center Groningen, University of Groningen, Groningen, The Netherlands, 9700RB.; 44 MRC Functional Genomics Unit, Department of Physiology, Anatomy & Genetics, Oxford University, UK; 45 Nuffield Department of Clinical Neuroscience, University of Oxford, UK; 46 Oxford Headache Centre, John Radcliffe Hospital, Oxford, UK; 47 Max-Planck-Institute of Psychiatry, Munich, Germany; 48 Christian Albrechts University, Kiel, Germany; 49 Institute of Human Genetics, Helmholtz Center Munich, Neuherberg, Germany; 50 Department of General Practice and Primary Health Care, University of Helsinki and Helsinki University Hospital, Helsinki Finland; 51 National Institute for Health and Welfare, Helsinki, Finland; 52 Institute of Clinical Medicine, University of Helsinki, Helsinki, Finland; 53 Department of Environmental Health, Harvard T.H. Chan School of Public Health,Boston, USA 02115; 54 Dept of Internal Medicine, Erasmus University Medical Center, Rotterdam, the Netherlands, 3015 CN.; 55 Dept of Pain Management and Research, Oslo University Hospital, Oslo, 0424 Oslo, Norway; 56 Medical Faculty, University of Oslo, Oslo, 0318 Oslo, Norway; 57 Division of Mental Health, Norwegian Institute of Public Health,P.O. Box 4404 Nydalen, Oslo, Norway, NO-0403; 58 Kiel Pain and Headache Center, 24149 Kiel, Germany.; 59 Danish Headache Center, Department of Neurology, Rigshospitalet, Glostrup Hospital, University of Copenhagen, Denmark; 60 Institute of Biological Psychiatry, Mental Health Center Sct. Hans, University of Copenhagen, Roskilde, Denmark; 61 Institute Of Biological Psychiatry, MHC Sct. Hans, Mental Health Services Copenhagen, DK-2100 Copenhagen, Denmark; 62 Institute of Clinical Sciences, Faculty of Medicine and Health Sciences, University of Copenhagen, DK-2100 Copenhagen, Denmark; 63 iPSYCH - The Lundbeck Foundation's Initiative for Integrative Psychiatric Research, DK-2100 Copenhagen, Denmark; 64 Oslo University Hospital and University of Oslo, Oslo, Norway; 65 University of Barcelona, Barcelona, Spain; 66 Language and Genetics Department, Max Planck Institute for Psycholinguistics, Nijmegen, 6525 XD, The Netherlands; 67 Institute of Health and Biomedical Innovation, Queensland University of Technology, Brisbane, Australia; 68 National Institute on Aging, Bethesda, USA; 69 School of Medicine, University of Tampere, Kalevantie; 70 University Medical Center Hamburg-Eppendorf, Hamburg, Germany; 71 Karolinska Institutet, Stockholm, Sweden; 72 VU University, Amsterdam, The Netherlands; 73 Universitat Autònoma de Barcelona, Barcelona, Spain; 74 Sutter Health, Sacramento, USA; 75 Department of Public Health, University of Helsinki, Helsinki, Finland; 76 Department of Health, National Institute for Health and Welfare, Helsinki, Finland; 77 Research Centre of Applied and Preventive Cardiovascular Medicine, University of Turku, Turku, Finland, 20521; 78 Department of Clinical Physiology and Nuclear Medicine, Turku University Hospital, Turku, Finland, 20521; 79 Dept of Neurology, Erasmus University Medical Center, Rotterdam, the Netherlands, 3015 CN.; 80 Imperial College London, Department of Epidemiology and Biostatistics, MRC Health Protection Agency (HPE) Centre for Environment and Health, School of Public Health, UK, W2 1PG; 81 University of Oulu, Biocenter Oulu, Finland, Box 5000, Fin-90014 University of Oulu; 82 Oulu University Hospital, Unit of Primary Care, Oulu, Finland, Box 10, Fin-90029 OYS; 83 University Medical Center Hamburg Eppendorf, Institute of Human Genetics, 20246 Hamburg, Germany; 84 Population Health Research Institute, St George's, University of London, Cranmer Terrace, London SW17 0RE, UK; 85 Munich Cluster for Systems Neurology (SyNergy), Munich, Germany; 86 Leiden University Medical Centre, Department of Human Genetics, Leiden, The Netherlands, PO Box 9600, 2300 RC; 87 Faculty of Medicine, University of Iceland, 101 Reykjavik, Iceland; 88 Statistical and Genomic Epidemiology Laboratory, Institute of Health and Biomedical Innovation, Queensland University of Technology, 60 Musk Ave, Kelvin Grove, QLD 4059, Australia; 89 Department of Neurology, Massachusetts General Hospital, Boston, USA

**Keywords:** Genetic correlation, Mendelian randomization, migraine, headache

## Abstract

**Background:**

Nearly a fifth of the world’s population suffer from migraine headache, yet risk factors for this disease are poorly characterized.

**Methods:**

To further elucidate these factors, we conducted a genetic correlation analysis using cross-trait linkage disequilibrium (LD) score regression between migraine headache and 47 traits from the UK Biobank. We then tested for possible causality between these phenotypes and migraine, using Mendelian randomization. In addition, we attempted replication of our findings in an independent genome-wide association study (GWAS) when available.

**Results:**

We report multiple phenotypes with genetic correlation (*P*  < 1.06 × 10^−3^) with migraine, including heart disease, type 2 diabetes, lipid levels, blood pressure, autoimmune and psychiatric phenotypes. In particular, we find evidence that blood pressure directly contributes to migraine and explains a previously suggested causal relationship between calcium and migraine.

**Conclusions:**

This is the largest genetic correlation analysis of migraine headache to date, both in terms of migraine GWAS sample size and the number of phenotypes tested. We find that migraine has a shared genetic basis with a large number of traits, indicating pervasive pleiotropy at migraine-associated loci.

**Key Messages**

- We replicate previous findings that heart disease, lipid levels, blood pressure, autoimmune and psychiatric phenotypes are genetically correlated with migraine.
- We report a novel genetic correlation between type 2 diabetes and migraine.
- Diastolic blood pressure is both genetically correlated and potentially causal for migraine.

## Introduction

Migraine headache is the most common neurological disorder, affecting 15–20% of people over the course of their lifetimes.^1^ It is characterized as a severe headache, often accompanied by visual disturbances, nausea or sensitivity to stimuli. The presence of these visual disturbances defines two migraine subtypes: with and without aura. Recent developments in migraine treatment show promise, but still have limited efficacy.^2^ For these reasons, migraine is the most disabling neurological disease,^1^^,^^3^ motivating the need for a better understanding of its biology.

Using genetics to improve our knowledge of the disease is promising, as migraine is approximately 42% heritable.^4^ A recent genome-wide association study (GWAS) meta-analysis for migraine combined data from 23andMe Inc. and the International Migraine Headache Genetics Consortium, resulting in a combined sample size of 59 674 cases and 316 078 controls. This GWAS identified 38 loci associated with migraine headache.^5^ However, the biological mechanisms at these loci are not fully understood.

Identification of traits that are genetically correlated with—or causally related to—migraine could contribute to the understanding of the disease and suggest directions for possible therapeutics. Most earlier studies suggesting associations between migraine and various biomarkers are observational, which can suffer from confounding. Randomized controlled studies could help disentangle correlation from causation, but it is infeasible to screen dozens of biomarkers at scale. In contrast, human genetics data can be used to screen a large number of traits, suggesting phenotypes worthy of additional examination, and potentially identifying the randomized controlled trials that have the best chance of success.

Using human genetics data, we conducted two types of analyses to identify potential biomarkers that may play a role in migraine. The first approach, cross-trait linkage disequilibrium (LD) score regression, uses association statistics from genetic variants across the genome to estimate the genetic correlation between two traits of interest.^6^ The second, Mendelian randomization (MR), compares the effect of variants strongly associated with an exposure of interest with their association with a disease endpoint of interest (here, migraine). Under certain assumptions, these data can be used to estimate a causal effect of the exposure on the outcome.^7^

Previous studies have applied these approaches to study migraine. One study calculated genetic correlation between migraine and 42 other phenotypes, using data on migraine occurrence from 53 000 cases and 231 000 controls from 23andMe.^8^ They found evidence of genetic correlation between migraine and eight different traits, using cross-trait LD score regression. In addition, they found evidence of shared genetic variants influencing migraine and 15 traits they tested. However, this report did not apply conventional Mendelian randomization techniques for hypothesis testing. Furthermore, a larger migraine GWAS is now available, allowing for higher-powered replication of previous findings.

Therefore, we performed cross-trait LD score regression and Mendelian randomization (MR) between migraine and the 47 phenotypes comprising a recent GWAS release of multiple traits in UK Biobank. These traits include cardiovascular, blood, anthropomorphic, education, reproductive and neuropsychiatric phenotypes with significant heritability and polygenicity, making them suitable for genetic correlation analyses.^9^ When possible, we sought to replicate genetic correlations with a *P*-value < 1.06 × 10^−^^3^, corresponding to a Bonferroni correction to 0.05 for the number of traits, in an independent GWAS. In addition, we analysed several additional phenotypes that have previously been associated with migraine: Alzheimer’s, serum calcium, serum magnesium and serum vitamin D levels.

## Methods

### Data

We obtained migraine data from the International Migraine Headache Genetics Consortium. For migraine-all, this GWAS is a meta-analysis of 22 different cohorts.^5^ For migraine with aura and migraine without aura, the GWAS excluded the 23andMe cohort, leading to a smaller sample size for the migraine subtypes.

We obtained the UK Biobank GWAS from [https://data.broadinstitute.org/alkesgroup/UKBB/]. We used the effective sample sizes (N_eff_) provided with the data for these traits.

For our calcium analysis, we could only obtain genome-wide calcium summary statistics for the discovery analysis from O’Seaghdha *et al*.^26^ However, summary statistics for the meta-analysis from O’Seaghdha are available for their lead single nucleotide polymorphisms (SNPs). Therefore, we used the statistics from the meta-analysis for our calcium MR, and used the discovery analysis statistics for calcium at each of the diastolic blood pressure instrumental variables in the multivariable MR analysis.

The data from Lee *et al*. we used excluded 23andMe due to data sharing issues.^20^

### Mendelian randomization

We performed our Mendelian randomization analyses using the MR-base R package.^48^ We generated the instrumental variables for each BOLT-LMM GWAS trait using the clump_data feature with default parameters, and filtered out SNPs with INFO scores below 0.9. For the remaining GWAS, we obtained instruments using plink’s clump_data function with a R^2^ < 0.001.^51^ For the MR-PRESSO analysis, we used the same instruments as in our standard MR analysis. We used default parameters, except we increased the NbDistribution parameter until MR-PRESSO could compute empirical *P*-values.^14^

### Cross-trait LD score regression

We performed cross-trait LD score regression using the linkage disequilibrium score (LDSC) regression package with default parameters.^6^ SNP info scores were used, when available, to filter for high-quality variants, and the y-intercept was left unconstrained.

## Results

### Widespread genetic correlation with migraine headache

**Figure 1:**
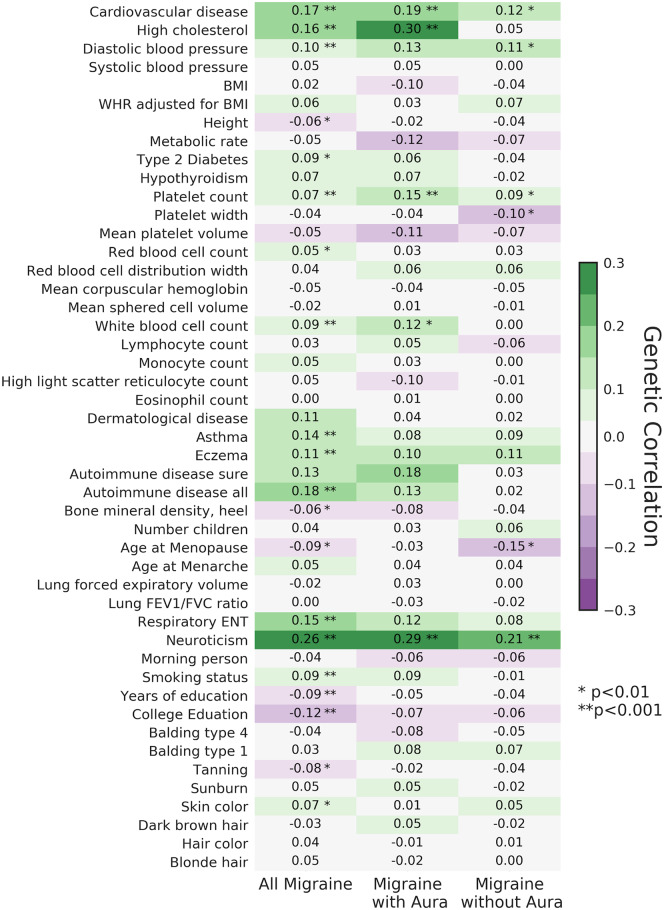
Cross-trait linkage disequilibrium score regression results between migraine and 47 different phenotypes from the UK Biobank. Numbers correspond to the strength of genetic correlation, and asterisks represent *P*-values of these associations. BMI, body mass index; FEV1, forced expiratory volume in 1 s; FVC, forced vital capacity; ENT, ear, nose and throat disorders.

To identify traits which may share a genetic basis with migraine, we first performed a large-scale, cross-trait genetic correlation analysis, using the framework of cross-trait LD score regression.^6^ Overall, we identified 14 traits from the UK Biobank (UKB) GWAS with genetic correlations with migraine, including cardiovascular disease, blood pressure, cholesterol, blood pressure, neuroticism, asthma, autoimmune disease, education, white blood cell count, platelet count and smoking status (Figure 1; Supplementary Table 1, available as Supplementary data at *IJE* online). In what follows, we describe these results in further detail, including the results of replication experiments. We also include results for each trait category for Mendelian randomization. We perform these analyses on all subjects with migraine (all subtypes), migraine with aura and migraine without aura.

### Genetic correlation between migraine and cardiovascular phenotypes

We began by examining migraine and cardiovascular disease and related traits. First, for the endpoint of cardiovascular disease, we observed a strong genetic correlation with migraine-all [genetic correlation (r_g_) = 0.17, *P* = 8.4 × 10^−^^13^, Figure 1; Supplementary Table 1, available as Supplementary data at *IJE* online], as well as both subsets of migraine: with (r_g_=0.19, *P* = 4.0 × 10^−^^4^) and without (r_g_ = 0.12, *P *= 3.6 × 10^−^^3^) aura. Included in this cardiovascular disease grouping definition from the UKB are multiple phenotypes, including hypertension, stroke, high cholesterol and ischaemic attack (see the UKB trait definition given in Supplementary Table 2, available as Supplementary data at *IJE* online). The results of Mendelian randomization analyses using genetic liability to cardiovascular disease in UKB as the exposure were mixed between different methods (Supplementary Tables 3, 4 and 5, available as Supplementary data at *IJE* online).

We next sought to tease apart which phenotypes were driving this correlation. We first evaluated the genetic correlation between coronary artery disease and migraine, using association data from the CARDIOGram+C4D consortium.^10^ However, the genetic correlation between heart disease and migraine resulted in a *P*-value of 0.61 (Supplementary Table 6, available as Supplementary data at *IJE* online). We next calculated the genetic correlation between stroke and migraine, using association data from the MEGASTROKE consortium^11^ which included a general stroke category in addition to four different subcategories. Genetic correlation with stroke resulted in a *P*-value of 0.25 (Supplementary Table 6, available as Supplementary data at *IJE* online). These analyses suggest that cardiovascular-related traits, like blood pressure or lipids levels, rather than the specific disease endpoints, may be driving the genetic correlation observed in the UKB analysis.

We then turned to evaluate genetic correlation between measurements of blood pressure and migraine. We found a compelling genetic correlation between diastolic blood pressure and migraine (r_g_=0.1, *P* = 5.4 × 10^−^^5^, Figure 1; Supplementary Table 1, available as Supplementary data at *IJE* online) with nominal significance in migraine with and without aura. We attempted replication of the genetic correlation finding in a meta-analysis of GWAS of blood pressure, which included individuals from the Million Veterans Project and the International Consortium of Blood Pressure (ICBP).^12^ We observed replication with diastolic blood pressure (r_g_=0.11, *P* = 1.90 × 10^−^^6^), and a weaker effect with systolic blood pressure (r_g_ = 0.063, *P* = 0.011), supporting the hypothesis that blood pressure and migraine share a genetic basis in common (Supplementary Table 7, available as Supplementary data at *IJE* online).

Next, we applied Mendelian randomization to test the hypothesis that genetic elevation in blood pressure increases susceptibility to migraine. We observed that one standard deviation (1-SD) genetic elevation in diastolic blood pressure increased risk to migraine-all by 14% [odds ratio (OR) = 1.14, 95% confidence interval (CI) = 1.07–1.21, *P* = 8.9 × 10^−^^5^], and a 1-SD genetic elevation in systolic blood pressure increased risk to migraine-all by 9% (OR = 1.09, CI = 1.01–1.16, *P* = 0.018) (Supplementary Table 3, available as Supplementary data at *IJE* online). Supporting this observation are all five Mendelian randomization methods estimating a positive effect estimate for both diastolic and systolic blood pressure on migraine, with the exception of one: weighted mode with systolic blood pressure (OR = 0.98, *P* = 0.79). We did not attempt replication of the Mendelian randomization effect, because the considerable cohort overlap between the ICBP and migraine cohorts can bias Mendelian randomization effect estimates.^13^^,^^14^ We next used a Steiger directionality test and observed that the correct direction of effect was indeed genetically determined diastolic blood pressure affecting migraine (Supplementary Table 7, available as Supplementary data at *IJE* online).^15^

**Figure 2:**
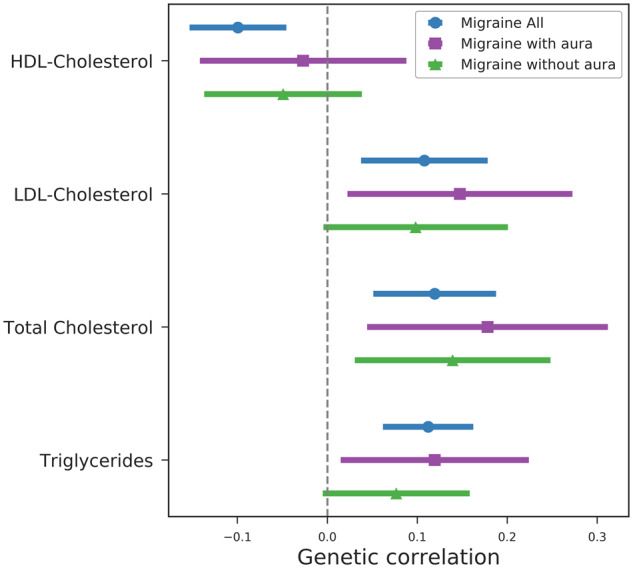
Genetic correlation of lipid traits with migraine headache and migraine subtypes using cross-trait linkage disequilibrium score regression. Error bars represent the 95% confidence interval. Lipid genome-wide association study is from Klarin (2018). HDL, high-density lipoprotein; LDL, low-density lipoprotein.

We subsequently turned to evaluating a role of plasma lipid levels in migraine. We observed strong genetic correlation between high cholesterol and migraine (all) and migraine with aura in the UK Biobank data (migraine-all: r_g_ = 0.16, *P* = 2.0 × 10^−^^6^; with-aura: r_g_ = 0.30, *P* = 1.3 × 10^−^^5^, Figure 1; Supplementary Table 1, available as Supplementary data at *IJE* online). We then tested for replication in an independent lipid GWAS meta-analysis of European individuals from the Millions Veterans Project and the Global Lipid Genetics Consortium.^12^ All four lipid traits [high-density lipoprotein (HDL) cholesterol, low-density lipoprotein (LDL) cholesterol, total cholesterol and triglyceride levels] reached our significance threshold, with triglycerides being the strongest (r_g_ = 0.11, *P *= 7.80 × 10^−^^6^, Figure 2; Supplementary Table 8, available as Supplementary data at *IJE* online). However, none of the Mendelian randomization experiments for the high cholesterol phenotype from UK Biobank or any of the four lipid phenotypes from the lipid GWAS meta-analysis had *P*-value less than 0.05 (Supplementary Table 8, available as Supplementary data at *IJE* online).

Finally, we looked for evidence of genetic correlation between adiposity traits and migraine, as many of these traits also relate to cardiovascular risk. In the UK Biobank data, we did not observe convincing genetic correlation between body mass index and migraine, and only a weak correlation with waist-hip ratio adjusted for body mass index (BMI) (Figure 1; Supplementary Table 1, available as Supplementary data at *IJE* online). Mendelian randomization results indicated that genetic elevation of these traits is not obviously associated with migraine (Supplementary Tables 3, 4 and 5, available as Supplementary data at *IJE* online).

### Genetic correlation between migraine and type-2 diabetes

We next looked for evidence of a shared genetic basis between type-2 diabetes (T2D) in the UKB and migraine. LD score regression suggests a positive genetic correlation between T2D and migraine (r_g_ = 0.09, *P* = 0.004) (Figure 1; Supplementary Table 1, available as Supplementary data at *IJE* online). Replication using a recently reported large-scale genetic association study for T2D, which included UKB individuals, resulted in an even stronger correlation (r_g_=0.11, *P* = 8.4 × 10^−^^5^, Supplementary Table 1, available as Supplementary data at *IJE* online).^16^ Mendelian randomization did not provide evidence for genetic elevation in T2D risk increasing risk of migraine (Supplementary Tables 3, 4 and 5, available as Supplementary data at *IJE* online).

### Genetic correlation between migraine and autoimmune-related and respiratory traits

We next explored the genetic relationship between autoimmune-related traits and migraine. There was a strong genetic correlation with an autoimmune phenotype category which encompasses a wide set of proposed autoimmune phenotypes in the UKB GWAS (r_g_=0.18, *P* = 2.7 × 10^−^^7^) (Supplementary Tables 1 and 2, available as Supplementary data at *IJE* online). In addition, a more specific set of autoimmune traits, denoted as ‘sure-autoimmune diseases’ (which included but was not limited to: type 1 diabetes, multiple sclerosis, lupus, Sjogren’s disease, coeliac disease and rheumatoid arthritis) were associated (r_g_=0.13, *P* = 0.012). Several additional diseases thought to have an autoimmune component also had genetic correlations with migraine, including eczema (r_g_ = 0.11, *P* = 6.9 × 10^−^^5^), respiratory and ear-nose-throat disease (r_g_=0.15, *P* = 4.6 × 10^−^^6^) and asthma (r_g_ = 0.14, *P* = 1.8 × 10^−^^5^, Figure 1; Supplementary Table 1, available as Supplementary data at *IJE* online).

We then attempted replication of these genetic correlations. Using two different asthma GWAS, asthma was associated with migraine with a stronger *P*-value (r_g_ = 0.17 and 0.11, *P* = 2.9 × 10^−^^7^ and 0.01) (Supplementary Table 9, available as Supplementary data at *IJE* online).^17^^,^^18^ We next attempted replication of the eczema association.^19^ The direction of effect remained consistent but the effect was less significant (r_g_ = 0.11, *P* = 0.07) (Supplementary Table 9, available as Supplementary data at *IJE* online). Mendelian randomization analyses were not suggestive for any of these trait categories (Supplementary Tables 3, 4 and 5, available as Supplementary data at *IJE* online).

### Genetic correlation between migraine and psychiatric and educational attainment traits

Next, we measured the genetic correlation between education level and migraine. We observed an inverse genetic correlation between migraine and both years of education (r_g_ = −0.09, *P* = 2.0 × 10^−^^5^) and having a college or university degree (r_g_ = −0.12, *P* = 1.1 × 10^−^^9^) (Figure 1; Supplementary Table 1, available as Supplementary data at *IJE* online). Replication of the genetic correlation in the largest GWAS for educational attainment to date was strong (r_g_ = −0.11, *P* = 1.9 × 10^−^^8^) (Supplementary Table 10, available as Supplementary data at *IJE* online).^20^ We next tested for a genetic association between cognitive performance and migraine using LD score regression but did not observe association (Supplementary Table 10, available as Supplementary data at *IJE* online).^20^ We note that a lack of genetic correlation with cognitive performance could be due to a difference in discovery power because of sample size (*n* = 766 345 for educational attainment, versus *n *= 257 828 for cognitive performance). Although Mendelian randomization for college education and years of education using the inverse variance weighted method was positive, neither of these results was robust in subsequent sensitivity analyses (Supplementary Table 3, available as Supplementary data at *IJE* online).

We next examined the genetic correlation between psychiatric traits and migraine. There was a strong, positive genetic correlation between all three migraine types and neuroticism both in UKB (r_g_ = 0.26, *P* = 5.8 × 10^−^^27^ with migraine-all, Figure 1) and in a higher-powered GWAS, which includes a UKB cohort (r_g_ = 0.26, *P* = 6.5 × 10^−^^28^).^21^ We saw genetic correlation among the two neuroticism subtypes as well: depressed affect (r_g_ = 0.30, *P* = 1.4 × 10^−^^28^) and worry (r_g_ = 0.21, *P* = 4.0 × 10^−^^18^) (Supplementary Table 11, available as Supplementary data at *IJE* online).^22^ There was also genetic correlation between migraine and general depression (r_g_ = 0.30, *P* = 2.7 × 10^−^^22^) (Supplementary Table 11, available as Supplementary data at *IJE* online).^21^ In the UKB, Mendelian randomization demonstrated that a genetic elevation in neuroticism was associated with an increased risk of migraine, using most MR methodological approaches (OR = 1.09, CI = 1.05–1.13, *P* = 9.1 × 10^−^^6^) (Supplementary Table 3, available as Supplementary data at *IJE* online). In addition, the MR-PRESSO method did not detect instruments with heterogeneity of effects with *P*-value < 0.05 (Supplementary Table 12, available as Supplementary data at *IJE* online). We were unable to attempt to replicate this MR effect using the higher-powered GWAS of Nagel *et al.*^22^ as effect sizes and standard errors were not available. Instead, we performed a Mendelian randomization using the results of Okbay *et al*., which has a smaller sample size. We found that the effect did not replicate, which may not be surprising given that there were only 12 genome-wide significance associations (Supplementary Table 12, available as Supplementary data at *IJE* online).

### Genetic correlation between migraine and blood traits

In the UK Biobank GWAS set, we observed a genetic correlation of migraine-all with blood platelet count (r_g_ = 0.08, *P* = 3 × 10^−^^4^) and white blood cell count (r_g_= 0.09, *P* = 5.0 × 10^−^^5^) (Figure 1; Supplementary Table 1, available as Supplementary data at *IJE* online). We next conducted an exploratory analysis to determine if there were additional blood platelet traits correlated with migraine, using results from the combined INTERVAL and UKB cohorts.^23^ We found 10 blood traits overall, including platelet count and white blood cell count, with nominal evidence of correlation (*P* < 0.05, out of a total of 36 different traits) (Supplementary Figure 1, Supplementary Table 13, available as Supplementary data at *IJE* online). Mendelian randomization analyses between blood traits in UKB and migraine did not return convincing support for causal effects (Supplementary Table 3, available as Supplementary data at *IJE* online).

### Hypothesis testing of previously associated phenotypes

We next tested for association between migraine and phenotypes not present in the set of UKB GWAS we used. We first tested for association with Alzheimer’s disease, using a GWAS of 455 258 individuals.^24^ We found a genetic correlation between migraine and migraine with aura and Alzheimer’s (r_g_ = 0.18, *P* = 0.014 for migraine-all and r_g_ = 0.3, *P* = 0.02 for migraine with aura) (Supplementary Table 14, available as Supplementary data at *IJE* online). However, a follow-up of this analysis, using an Alzheimer’s GWAS composed of 94 437 individuals, did not support this finding (r_g_ = −0.034, *P* = 0.59 for migraine-all and r_g_ = −0.039, *P* = 0.75 for migraine with aura) (Supplementary Table 14, available as Supplementary data at *IJE* online).^25^

We next checked for a genetic correlation between migraine and biomarkers which have been previously hypothesized to be associated with migraine headache. Indeed, we found an association between serum calcium and migraine-all (r_g_ = 0.13, *P* = 0.017) using cross-trait LD score regression, and a directionally consistent effect of calcium on migraine using Mendelian randomization (OR = 1.51 *P* = 0.07) (Supplementary Table 15, available as Supplementary data at *IJE* online).^26^ We found no association between magnesium and migraine, using Mendelian randomization (Supplementary Table 16, available as Supplementary data at *IJE* online),^27^ and were unable to perform cross-trait LD score regression with magnesium because genome-wide summary data are not available. In addition, we found no genetic correlation between serum vitamin D levels and migraine (Supplementary Table 17, available as Supplementary data at *IJE* online).^28^

### Multivariable analysis of vascular traits

Our analyses find evidence for a potentially causal relationship between migraine and diastolic blood pressure, and provide modest replication of a calcium association that we previously reported.^29^ This leads to the question of whether these putative causal relationships are independent of one another. To answer this question, we first tested for genetic correlation between calcium and diastolic blood pressure using two different blood pressure GWAS: the UKB results and a combined meta-analysis comprising over 1 million individuals.^12^ We found a genetic correlation with *P*-values 0.003 and 0.0011 between diastolic and calcium levels using the two blood pressure GWAS (Supplementary Table 18, available as Supplementary data at *IJE* online), strengthening the hypothesis that blood pressure and calcium may not have independent causal effects on migraine.

**Figure 3:**
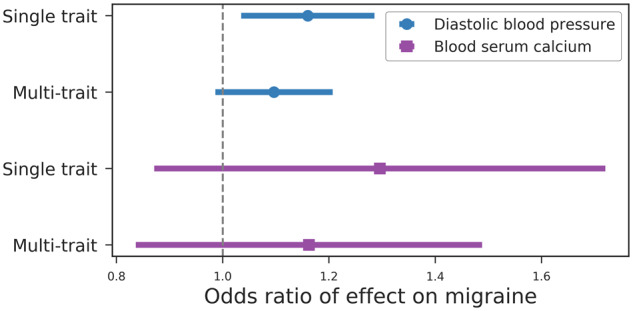
Effect of diastolic blood pressure and calcium on migraine-all. ‘Single trait’ is the estimated effect of the given biomarker on migraine-all using Mendelian randomization of only the given biomarker. “Multi-trait” is the estimated effect of the biomarker on migraine-all using the residual of the outcome after adjustment for the other biomarker. Error bars represent the 95% confidence interval.

To more thoroughly test this hypothesis, we performed multivariable MR, which considers the effects of several different exposures jointly. When fitting each exposure to the residual of the outcome adjusted for the other exposure, the effect of serum calcium levels on migraine-all was attenuated (odds ratio of 1.29 to 1.16), whereas the effect of diastolic blood pressure on migraine-all remained more similar (odds ratio 1.16 to 1.10) after inclusion of calcium in the model (Figure 3; Supplementary Table 18, available as Supplementary data at *IJE* online). We next tested whether diastolic blood pressure and calcium have a clear causal relationship. Mendelian randomization analyses between serum calcium and diastolic blood pressure, or the reciprocal diastolic blood pressure on serum calcium, were inconclusive (Supplementary Table 18, available as Supplementary data at *IJE* online). Heterogeneity between instruments, potentially driven by pleiotropy, could bias these results. To test for this, we performed an MR-PRESSO analysis^14^ which removes instruments demonstrating horizontal pleiotropy, and found that the diastolic blood pressure effect on migraine remained (*P* = 0.02) (Supplementary Table 18, available as Supplementary data at *IJE* online). No heterogeneity was detected between the calcium instruments.

## Discussion

Here, we report genome-wide correlations between migraine headache and a wide range of traits. We suspect that the sizeable number of correlations passing a conservative Bonferroni correction could be a result of the large sample size of both the UK Biobank and migraine GWAS, combined with the pleiotropic nature of variants contributing to migraine susceptibility. Our large number of reported correlations is consistent with previous studies of genetic correlation which include migraine.^8^^,^^30^ We note that although some of these correlations were only present for migraine-all or one migraine subtype, it is difficult to make claims about heterogeneity of effects between subtypes, given the reduced sample sizes of the subtype cohorts.

It is important to note that many of the phenotypes in the UK Biobank are influenced by the tendency of individuals to report a phenotype to a doctor. For instance, the observed genetic association between neuroticism and migraine could be due to neuroticism increasing the likelihood of reporting having had migraine to a doctor. In addition, we stress that two-sample Mendelian randomization, as used here, does not test for a causal effect of a disease on an outcome, but instead tests for a causal effect between genetic liability for the disease and an outcome.

We find pervasive evidence of genetic correlation between migraine and other brain-related traits. We report a novel genetic correlation between Alzheimer’s disease and migraine; however, this correlation did not successfully replicate when using a smaller GWAS. The lack of replication could be due to several factors, including the smaller sample size decreasing power, or the difference in case criteria: the larger GWAS of Jensen *et al*. included both clinically diagnosed Alzheimer’s patients and Alzheimer’s-by-proxy cases, which was based on parental diagnoses. The GWAS of Kunkle *et al*. only used clinically diagnosed cases. Consistent with our results, previous studies suggest an inverse correlation between educational attainment and migraine.^8^^,^^31–33^ We also find a positive genetic correlation between neuroticism and depression and migraine, matching earlier reports.^34–37^.

We find no evidence for a relationship between migraine and magnesium or vitamin D. Some studies have found support of these nutrients as a migraine preventative, but the evidence is limited.^38–40^ However, our analysis may lack statistical power: there were only eight independent genetic variants for magnesium and 10 for vitamin D. Currently, the role of blood platelet traits in migraine is not well understood; however, our findings corroborate those of Pickrell *et al.*, and suggest a shared genetic basis.^8^

We find evidence of genetic correlation between migraine and only some of the tested cardiometabolic traits, which is perhaps surprising given previous genetic and epidemiological observations. Pickrell *et al*. showed a shared genetic basis between heart disease and migraine, using data from the CARDIoGRAM+C4D consortium using a conjunction false-discovery rate (FDR) approach, which measures how much of an excess of significantly associated variants in one trait can be accounted for due to associations with a second trait.^8^ Observational studies have also found a correlation between the occurrence of these diseases.^41^ However, consistent with what we report here, Pickrell *et al.* found no genetic correlation using cross-trait LD score regression on their migraine dataset. One possible explanation for the discordance between these cross-trait LD score results and other types of evidence could be that heart disease is not pleiotropic enough for a high-powered genetic correlation analysis. An additional explanation is that coronary artery disease and migraine share only a subset of causal single nucleotide polymorphisms (SNPs), diluting the genetic correlation signal. Consistent with our results, lipid levels have been previously associated with migraine severity.^42^^,^^43^ We also observed a novel positive genetic correlation with type 2 diabetes. Previous observational studies have found an inconsistent correlation between migraine and type 2 diabetes, ^44–46^ with some evidence suggesting there may be an age-dependent effect.^44^ Our genetic correlation analysis provides evidence that there may be a shared genetic basis between these traits, which may be obscured in observational studies by environmental or pharmacological factors.

In addition, we report both a novel positive genetic correlation and a potential causative relationship between blood pressure and migraine. This contrasts with a recent study which found an inverse relationship between blood pressure and migraine and tension headache, but corroborates the observation that beta-blockers which lower blood pressure can decrease migraine attack frequency.^47^^,^^48^ However, to our knowledge, no large-scale observational study of blood pressure and migraine has been performed. Our multivariable analysis does not reveal a clear causal order between calcium, blood pressure and migraine, but suggests that calcium and blood pressure do not have strictly independent effects on migraine. However, these results do suggest that neurovascular processes associated with increased blood pressure may underlie migraine headache,^49^ supporting a recent study which found that migraine disease heritability is enriched in genes specifically expressed in cardiovascular tissues.^50^

These findings reveal potential shared biology between migraine and multiple other phenotypes. This motivates further work to reveal the genetic and functional basis of these observations, either through multi-trait association studies or through functional follow-up.

## Authors

### The International Headache Genetics Consortium

Padhraig Gormley*,^1,2,3,4^, Verneri Anttila*,^2,3,5^, Bendik S Winsvold^6,7,8^, Priit Palta^9^, Tonu Esko^2,10,11^, Tune H. Pers^2,11,12,13^, Kai-How Farh^2,5,14^, Ester Cuenca-Leon^1,2,3,15^, Mikko Muona^9,16,17,18^, Nicholas A Furlotte^19^, Tobias Kurth^20,21^, Andres Ingason^22^, George McMahon^23^, Lannie Ligthart^24^, Gisela M Terwindt^25^, Mikko Kallela^26^, Tobias M Freilinger^27,28^, Caroline Ran^29^, Scott G Gordon^30^, Anine H Stam^25^, Stacy Steinberg^22^, Guntram Borck^31^, Markku Koiranen^32^, Lydia Quaye^33^, Hieab HH Adams^34,35^, Terho Lehtimäki^36^, Antti-Pekka Sarin^9^, Juho Wedenoja^37^, David A Hinds^19^, Julie E Buring^21,38^, Markus Schürks^39^, Paul M Ridker^21,38^, Maria Gudlaug Hrafnsdottir^40^, Hreinn Stefansson^22^, Susan M Ring^23^, Jouke-Jan Hottenga^24^, Brenda WJH Penninx^41^, Markus Färkkilä^26^, Ville Artto^26^, Mari Kaunisto^9^, Salli Vepsäläinen^26^, Rainer Malik^27^, Andrew C Heath^42^, Pamela A F Madden^42^, Nicholas G Martin^30^, Grant W Montgomery^30^, Mitja Kurki^1,2,3^, Mart Kals^10^, Reedik Mägi^10^, Kalle Pärn^10^, Eija Hämäläinen^9^, Hailiang Huang^2,3,5^, Andrea E Byrnes^2,3,5^, Lude Franke^43^, Jie Huang^4^, Evie Stergiakouli^23^, Phil H Lee^1,2,3^, Cynthia Sandor^44^, Caleb Webber^44^, Zameel Cader^45,46^, Bertram Muller-Myhsok^47^, Stefan Schreiber^48^, Thomas Meitinger^49^, Johan G Eriksson^50,51^, Veikko Salomaa^51^, Kauko Heikkilä^52^, Elizabeth Loehrer^34,53^, Andre G Uitterlinden^54^, Albert Hofman^34^, Cornelia M van Duijn^34^, Lynn Cherkas^33^, Linda M. Pedersen^6^, Audun Stubhaug^55,56^, Christopher S Nielsen^55,57^, Minna Männikkö^32^, Evelin Mihailov^10^, Lili Milani^10^, Hartmut Göbel^58^, Ann-Louise Esserlind^59^, Anne Francke Christensen^59^, Thomas Folkmann Hansen^60^, Thomas Werge^61,62,63^, Sigrid Børte^64^, Bru Cormand^65^, Else Eising^66^, Lyn Griffiths^67^, Eija Hamalainen^9^, Marjo Hiekkala^16^, Risto Kajanne^9^, Lenore Launer^68^, Terho Lehtimaki^69^, Davor Leslsel^70^, Alfons Macaya^15^, Massimo Mangino^33^, Nancy Pedersen^71^, Danielle Posthuma^72^, Patricia Pozo-Rosich^73^, Alice Pressman^74^, Celia Sintas^65^, Marta Vila-Pueyo^15^, Huiying Zhao^68^. Jaakko Kaprio^9,75,76^, Arpo J Aromaa^51^, Olli Raitakari^77,78^, M Arfan Ikram^34,35,78^, Tim Spector^33^, Marjo-Riitta Järvelin^32,80,81,82^, Andres Metspalu^10^, Christian Kubisch^83^, David P Strachan^84^, Michel D Ferrari^25^, Andrea C Belin^29^, Martin Dichgans^27,85^, Maija Wessman^9,16^, Arn MJM van den Maagdenberg^25,86^, John-Anker Zwart^6,7,8^, Dorret I Boomsma^24^, George Davey Smith^23^, Kari Stefansson^22,87^, Nicholas Eriksson^19^, Mark J Daly^2,3,5^, Benjamin M Neale§,^2,3,5^, Jes Olesen^§,59^, Daniel I Chasman^§,21,38^, Dale R Nyholt^§,88^, and Aarno Palotie^§,1,2,3,4,5,9,89^.

^1^Psychiatric and Neurodevelopmental Genetics Unit, Massachusetts General Hospital and Harvard Medical School, Boston, USA. ^2^Medical and Population Genetics Program, Broad Institute of MIT and Harvard, Cambridge, USA. ^3^Stanley Center for Psychiatric Research, Broad Institute of MIT and Harvard, Cambridge, USA. ^4^Wellcome Trust Sanger Institute, Wellcome Trust Genome Campus, Hinxton, UK. ^5^Analytic and Translational Genetics Unit, Massachusetts General Hospital and Harvard Medical School, Boston, USA. ^6^FORMI, Oslo University Hospital, P.O. 4956 Nydalen, 0424 Oslo, Norway. ^7^Department of Neurology, Oslo University Hospital, P.O. 4956 Nydalen, 0424 Oslo, Norway. ^8^Institute of Clinical Medicine, University of Oslo, P.O. 1171 Blindern, 0318 Oslo, Norway. ^9^Institute for Molecular Medicine Finland (FIMM), University of Helsinki, Helsinki, Finland. ^10^Estonian Genome Center, University of Tartu, Tartu, Estonia. ^11^Division of Endocrinology, Boston Children's Hospital, Boston, USA. ^12^Statens Serum Institut, Dept of Epidemiology Research, Copenhagen, Denmark. ^13^Novo Nordisk Foundation Center for Basic Metabolic Research, University of Copenhagen, Copenhagen, Denmark. ^14^Illumina, 5200 Illumina Way, San Diego, USA. ^15^Vall d'Hebron Research Institute, Pediatric Neurology, Barcelona, Spain. ^16^Folkhälsan Institute of Genetics, Helsinki, Finland, FI-00290. ^17^Neuroscience Center, University of Helsinki, Helsinki, Finland, FI-00014. ^18^Research Programs Unit, Molecular Neurology, University of Helsinki, Helsinki, Finland, FI-00014. 1923andMe, Inc., 899 W. Evelyn Avenue, Mountain View, CA, USA. ^20^Inserm Research Center for Epidemiology and Biostatistics (U897), University of Bordeaux, 33076 Bordeaux, France. ^21^Division of Preventive Medicine, Brigham and Women's Hospital, Boston MA 02215. ^22^deCODE Genetics, 101 Reykjavik, Iceland. ^23^Medical Research Council (MRC) Integrative Epidemiology Unit, University of Bristol, Bristol, UK. ^24^VU University Amsterdam, Department of Biological Psychology, Amsterdam, the Netherlands, 1081 BT. ^25^Leiden University Medical Centre, Department of Neurology, Leiden, The Netherlands, PO Box 9600, 2300 RC. ^26^Department of Neurology, Helsinki University Central Hospital, Haartmaninkatu 4, 00290 Helsinki, Finland. ^27^Institute for Stroke and Dementia Research, Klinikum der Universtität München, Ludwig-Maximilians-Universität München, Feodor-Lynen-Str. 17, 81377 Munich Germany. ^28^Department of Neurology and Epileptology, Hertie Institute for Clincal Brain Research, University of Tuebingen. ^29^Karolinska Institutet, Department of Neuroscience, 171 77 Stockholm, Sweden. ^30^Department of Genetics and Computational Biology, QIMR Berghofer Medical Research Institute, 300 Herston Road, Brisbane, QLD 4006, Australia. ^31^Ulm University, Institute of Human Genetics, 89081 Ulm, Germany. ^32^University of Oulu, Center for Life Course Epidemiology and Systems Medicine, Oulu, Finland, Box 5000, Fin-90014 University of Oulu. ^33^Department of Twin Research and Genetic Epidemiology, King's College London, London, UK. ^34^Dept of Epidemiology, Erasmus University Medical Center, Rotterdam, the Netherlands, 3015 CN. ^35^Dept of Radiology, Erasmus University Medical Center, Rotterdam, the Netherlands, 3015 CN. ^36^Department of Clinical Chemistry, Fimlab Laboratories, and School of Medicine, University of Tampere, Tampere, Finland, 33520. ^37^Department of Public Health, University of Helsinki, Helsinki, Finland. ^38^Harvard Medical School, Boston MA 02115. ^39^University Duisburg Essen, Essen, Germany. ^40^Landspitali University Hospital, 101 Reykjavik, Iceland. ^41^VU University Medical Centre, Department of Psychiatry, Amsterdam, the Netherlands, 1081 HL. ^42^Department of Psychiatry, Washington University School of Medicine, 660 South Euclid, CB 8134, St. Louis, MO 63110, USA. ^43^University Medical Center Groningen, University of Groningen, Groningen, The Netherlands, 9700RB. ^44^MRC Functional Genomics Unit, Department of Physiology, Anatomy & Genetics, Oxford University, UK. ^45^Nuffield Department of Clinical Neuroscience, University of Oxford, UK. ^46^Oxford Headache Centre, John Radcliffe Hospital, Oxford, UK. ^47^Max-Planck-Institute of Psychiatry, Munich, Germany. ^48^Christian Albrechts University, Kiel, Germany. ^49^Institute of Human Genetics, Helmholtz Center Munich, Neuherberg, Germany. ^50^Department of General Practice and Primary Health Care, University of Helsinki and Helsinki University Hospital, Helsinki Finland. ^51^National Institute for Health and Welfare, Helsinki, Finland. ^52^Institute of Clinical Medicine, University of Helsinki, Helsinki, Finland. ^53^Department of Environmental Health, Harvard T.H. Chan School of Public Health, Boston, USA 02115. ^54^Dept of Internal Medicine, Erasmus University Medical Center, Rotterdam, the Netherlands, 3015 CN. ^55^Dept of Pain Management and Research, Oslo University Hospital, Oslo, 0424 Oslo, Norway. ^56^Medical Faculty, University of Oslo, Oslo, 0318 Oslo, Norway. ^57^Division of Mental Health, Norwegian Institute of Public Health,P.O. Box 4404 Nydalen, Oslo, Norway, NO-0403. ^58^Kiel Pain and Headache Center, 24149 Kiel, Germany. ^59^Danish Headache Center, Department of Neurology, Rigshospitalet, Glostrup Hospital, University of Copenhagen, Denmark. ^60^Institute of Biological Psychiatry, Mental Health Center Sct. Hans, University of Copenhagen, Roskilde, Denmark. ^61^Institute Of Biological Psychiatry, MHC Sct. Hans, Mental Health Services Copenhagen, DK-2100 Copenhagen, Denmark. ^62^Institute of Clinical Sciences, Faculty of Medicine and Health Sciences, University of Copenhagen, DK-2100 Copenhagen, Denmark. ^63^iPSYCH - The Lundbeck Foundation's Initiative for Integrative Psychiatric Research, DK-2100 Copenhagen, Denmark. ^64^Oslo University Hospital and University of Oslo, Oslo, Norway. ^65^University of Barcelona, Barcelona, Spain. ^66^Language and Genetics Department, Max Planck Institute for Psycholinguistics, Nijmegen, 6525 XD, The Netherlands. ^67^Institute of Health and Biomedical Innovation, Queensland University of Technology, Brisbane, Australia. ^68^National Institute on Aging, Bethesda, USA. ^69^School of Medicine, University of Tampere, Kalevantie. ^70^University Medical Center Hamburg-Eppendorf, Hamburg, Germany. ^71^Karolinska Institutet, Stockholm, Sweden. ^72^VU University, Amsterdam, The Netherlands. ^73^Universitat Autònoma de Barcelona, Barcelona, Spain. ^74^Sutter Health, Sacramento, USA. ^75^Department of Public Health, University of Helsinki, Helsinki, Finland. ^76^Department of Health, National Institute for Health and Welfare, Helsinki, Finland. ^77^Research Centre of Applied and Preventive Cardiovascular Medicine, University of Turku, Turku, Finland, 20521. ^78^Department of Clinical Physiology and Nuclear Medicine, Turku University Hospital, Turku, Finland, 20521. ^79^Dept of Neurology, Erasmus University Medical Center, Rotterdam, the Netherlands, 3015 CN. ^80^Imperial College London, Department of Epidemiology and Biostatistics, MRC Health Protection Agency (HPE) Centre for Environment and Health, School of Public Health, UK, W2 1PG. ^81^University of Oulu, Biocenter Oulu, Finland, Box 5000, Fin-90014 University of Oulu. ^82^Oulu University Hospital, Unit of Primary Care, Oulu, Finland, Box 10, Fin-90029 OYS. ^83^University Medical Center Hamburg Eppendorf, Institute of Human Genetics, 20246 Hamburg, Germany. ^84^Population Health Research Institute, St George's, University of London, Cranmer Terrace, London SW17 0RE, UK. ^85^Munich Cluster for Systems Neurology (SyNergy), Munich, Germany. ^86^Leiden University Medical Centre, Department of Human Genetics, Leiden, The Netherlands, PO Box 9600, 2300 RC. ^87^Faculty of Medicine, University of Iceland, 101 Reykjavik, Iceland. ^88^Statistical and Genomic Epidemiology Laboratory, Institute of Health and Biomedical Innovation, Queensland University of Technology, 60 Musk Ave, Kelvin Grove, QLD 4059, Australia. ^89^Department of Neurology, Massachusetts General Hospital, Boston, USA.

* These authors contributed equally to this work. § These authors jointly supervised this work.

### Collaborators

The 23andMe research Team

## Supplementary Data

Supplementary data are available at *IJE* online.

## Funding

This work was supported by US National Institutes of Health (DK101478 and HG010067 to B.F.V., T32 HG000046 for K.M.S.), and a Linda Pechenik Montague Investigator award (to B.F.V.). This research is based on data from the Million Veteran Program, Office of Research and Development, Veterans Health Administration, and was supported by award #MVP000. This publication does not represent the views of the Department of Veterans Affairs or of the United States Government. This research was also supported by two additional Department of Veterans Affairs awards (I01 BX003362 [P.S.T./K-M.C.], IK2-CX001780 [S.M.D.).

## Acknowledgements

We would like to acknowledge the members of the Voight Lab for their helpful feedback. In addition, we would like to thank the research participants and employees of 23andMe for making this work possible.

## Conflict of Interest

None declared.

## Supplementary Material

dyaa050_Supplementary_Data
