# Erratum to: Cross-trait analyses with migraine reveal widespread pleiotropy and suggest a vascular component to migraine headache

**DOI:** 10.1093/ije/dyaa154

**Published:** 2020-12-17

**Authors:** Katherine M Siewert, Derek Klarin, Scott M Damrauer, Kyong-Mi Chang, Philip S Tsao, Themistocles L Assimes, George Davey Smith, Benjamin F Voight

The International Headache Genetics Consortium was omitted from the Author list and the list of additional authors and collborators was published as a supplementary file.

The author list has now been corrected and members of The International Headache Genetics Consortium are listed as authors in the list of authors and collaborators at the end of the paper.

## Notes

First published online: 19 April 2020, *Int J Epidemiol* 2020;**49**:1022–31, doi: https://doi.org/10.1093/ije/dyaa050

