# Supplementary material for: Cross-trait analyses with migraine reveal widespread pleiotropy and suggest a vascular component to migraine headache
Source: Int J Epidemiol. 2020 Apr 19;49(3):1022–31. doi: 10.1093/ije/dyaa050 (PMC7394956; doi:10.1093/ije/dyaa050)
Supplement: dyaa050_Supplementary_Data [file dyaa050_supplementary_data.zip › dyaa050_Supplementary_Data/ije-2019-08-1138-Supplementary material.docx]

*
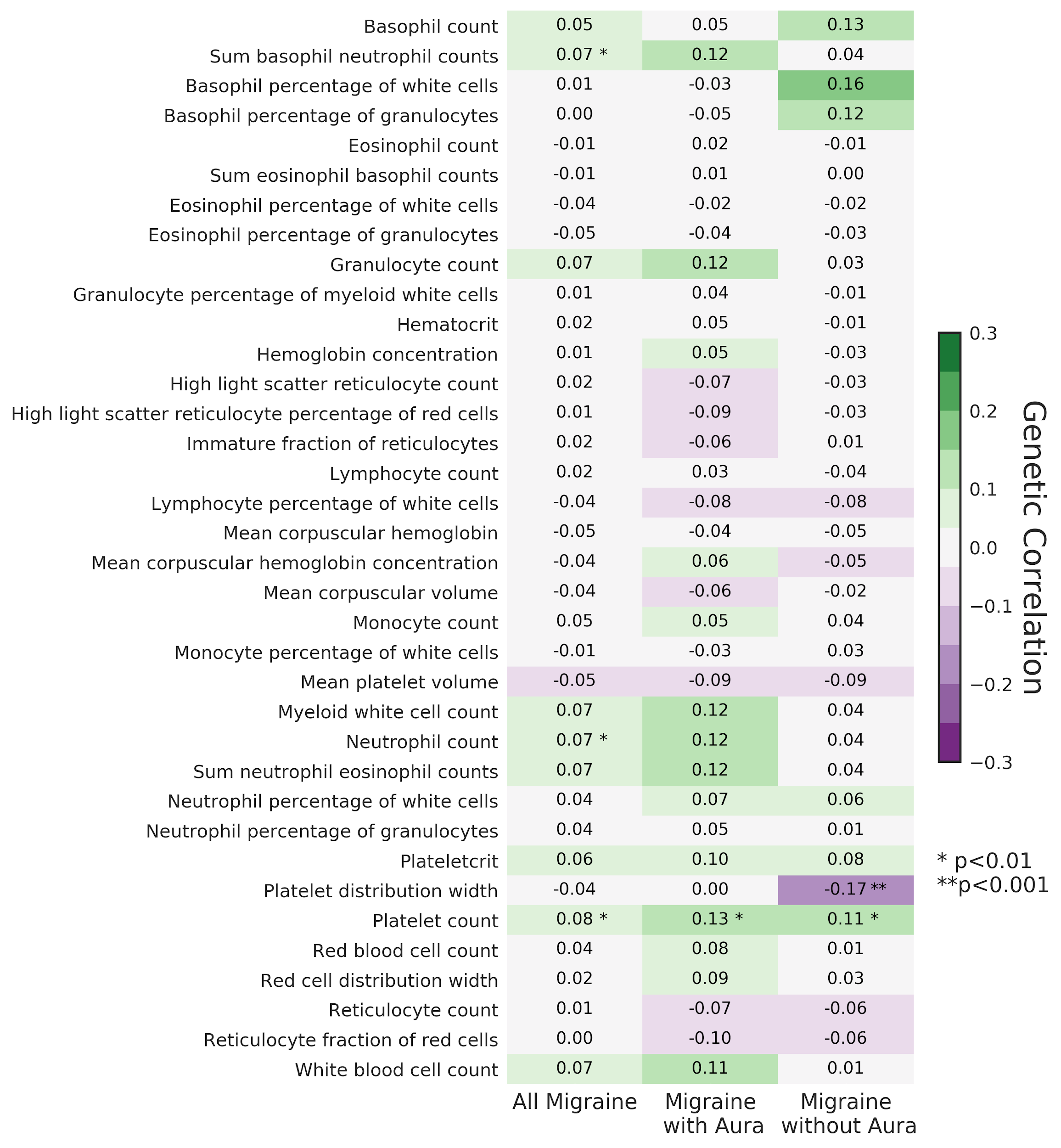
*

**Supplementary Figure 1:** Cross-trait LD score regression results between Migraine and blood phenotypes from Astle et al.
